# Identifying special operative trainees at-risk for musculoskeletal injury using full body kinematics

**DOI:** 10.3389/fbioe.2023.1293923

**Published:** 2023-12-06

**Authors:** Lance Frazer, Tylan Templin, Travis David Eliason, Cody Butler, Ben Hando, Daniel Nicolella

**Affiliations:** ^1^ Southwest Research Institute (SwRI), San Antonio, TX, United States; ^2^ United States Air Force, Special Warfare Training Wing Research Flight, Joint Base San Antonio-Lackland, San Antonio, TX, United States; ^3^ Kennell and Associates Inc, Falls Church, VA, United States

**Keywords:** biomechanics, military, functional movement analysis, kinematics, markerless motion capture, injury risk assessment

## Abstract

**Introduction:** Non-combat musculoskeletal injuries (MSKIs) during military training significantly impede the US military’s functionality, with an annual cost exceeding $3.7 billion. This study aimed to investigate the effectiveness of a markerless motion capture system and full-body biomechanical movement pattern assessments to predict MSKI risk among military trainees.

**Methods:** A total of 156 male United States Air Force (USAF) airmen were screened using a validated markerless biomechanics system. Trainees performed multiple functional movements, and the resultant data underwent Principal Component Analysis and Uniform Manifold And Projection to reduce the dimensionality of the time-dependent data. Two approaches, semi-supervised and supervised, were then used to identify at-risk trainees.

**Results:** The semi-supervised analysis highlighted two major clusters with trainees in the high-risk cluster having a nearly five times greater risk of MSKI compared to those in the low-risk cluster. In the supervised approach, an AUC of 0.74 was produced when predicting MSKI in a leave-one-out analysis.

**Discussion:** The application of markerless motion capture systems to measure an individual’s kinematic profile shows potential in identifying MSKI risk. This approach offers a novel way to proactively address one of the largest non-combat burdens on the US military. Further refinement and wider-scale implementation of these techniques could bring about substantial reductions in MSKI occurrence and the associated economic costs.

## Introduction

Non-combat musculoskeletal injuries (MSKIs) that occur during basic and specialized military training continue to be one of the greatest burdens affecting the United States military and their allied partners (Molloy et al., 2020; Rhon et al., 2022). The economic consequences and lost duty days are a substantial encumbrance on the functionality of our armed forces. Recent studies estimate that non-combat injuries are six times more likely than combat-related injuries, put 68,000 servicemembers in non-deployable status every year, and carry an annual cost that exceeds $3.7 billion (Grimm et al., 2019). Among MSKI types, lower extremity (LE) injuries are the most common with some reports attributing more than 70% of non-combat injuries to lower extremities (Molloy et al., 2020; Butler et al., 2022). As a result, the US military has promoted the identification of Service members at risk for LE MSKI and the development of effective preventative measures as a top research priority (Teyhen et al., 2020; Rhon et al., 2021).

Over the past decade, a growing interest has developed in the use of biomechanics to evaluate MSKI risk during military training (Sammito et al., 2021). Biomechanical assessments typically include the analysis of an individual’s movement (kinematics) (Hando et al., 2021; Bird et al., 2022; Cameron et al., 2022; Eckard et al., 2022; Bird et al., 2023), force transmission through the body (kinetics) (Bird et al., 2022; Hando et al., 2022; Bird et al., 2023), and/or overall fitness (Tomes et al., 2020). These analyses have been fruitful in discovering biomechanical measures associated with injury. For example, Sharma et al. found that imbalanced foot pressure is predictive of medial tibia stress syndrome (shin splints) (Sharma et al., 2011). Step width during gait has been associated with tibial stress, which may affect stress fracture risk (Meardon and Derrick, 2014). Wan and Shan associated muscle mechanics with repetitive stress injuries (Wan and Shan, 2016). While these analyses, among others (Negus and Sih, 2016; Winkelmann et al., 2016; Garnock et al., 2018), have shown promise in identifying Service members at risk for MSKI, to date, there are no examples of successful adoption of biomechanical assessments in military units that have reduced the injury burden. A well-generalizable set of predictive variables and an accurate, reliable injury risk algorithm has yet to be demonstrated. Moreover, the time and effort required to perform biomechanical assessments limit their application at scale, which is particularly important for military applications. To address this latter concern, markerless motion capture technology has emerged as promising to substantially reduce the burden of high throughput testing.

Markerless motion capture systems are an attractive screening tool because of their operational simplicity and equivalence to marker-based systems (Perrott et al., 2017; Martinez et al., 2018; Mosier et al., 2018; Drazan et al., 2021). However, the few studies to investigate their use for injury risk assessments have yielded mixed results, and it is unclear if these systems provide meaningful information to predict MSKI. For example, Eckard et al. and Cameron et al. used results from a markerless motion capture system to compute the Landing Error Scoring System (LESS) and found a significant association between individual subjects’ jumping/landing characteristics and lower limb bone stress injury (Cameron et al., 2022; Eckard et al., 2022). While promising, the association was most meaningful among female trainees and may not be strong enough to make actionable decisions for both males and females (Eckard et al., 2022). Moreover, the findings were only applicable to a small percentage of participants who landed in a very particular way (Cameron et al., 2022). In contrast, Hando et al., as well as Bird et al. used markerless motion capture technology coupled with proprietary “scores” but found poor association with injury risk (Hando et al., 2021; Bird et al., 2023). While the ease of implementation offered by markerless systems has alleviated the concern of high throughput testing, the limited and even conflicting results have questioned their predictive value. Yet, even the studies with much more inclusive methods to perform biomechanical assessments have not yielded satisfactory predictive power (Sanchez-Santos et al., 2017; Rhon et al., 2018; Sammito et al., 2021). Thus, it remains challenging to make a convincing case for biomechanics to assess injury risk.

We believe the application of markerless motion capture and the subsequent analyses of the kinematics obtained from such systems have greater potential to identify MSKI risk than these prior studies suggest. The majority of studies, including those cited earlier, use univariate measures that are decided upon *a priori* and researchers must subjectively choose what may or may not be predictive of injury (Chorba et al., 2010; Lisman et al., 2013; Sefton et al., 2016; Markström et al., 2019; Sammito et al., 2021; Bird et al., 2022; Cameron et al., 2022; Eckard et al., 2022; Hando et al., 2022). Individuals perform specific movements through unique, coordinated patterns of time-dependent joint motions that cannot be adequately described with univariate measures (or even a collection of univariate measures). These unique patterns, or biomechanical fingerprints, that include multiple joint motions across the entire body may reveal subtle differences that are indicative of injury risk. Yet, to our knowledge, no study has investigated the detailed, high-fidelity, full-body kinematics to determine the injury risk of military trainees. With this approach, full, time-dependent joint motions across the entire body are analyzed using dimensionality reduction techniques without choosing beforehand which joints and/or key events are important. We hypothesize that considering the entirety of an individual’s kinematic profile is predictive of injury. Therefore, to test this hypothesis and address a gap in injury risk analysis amongst military trainees, the purpose of this study is to evaluate whether an individual’s biomechanical movement pattern is indicative of injury risk.

## Materials and methods

### Participants

United States Air Force (USAF) airmen (male) entering a Special Warfare (SW) 8-week preparatory course designed to ready trainees for the rigors of Special Warfare training served as participants in the study. Details of this course have been described in prior studies from our group (Hando et al., 2022). Trainees with active injuries were excluded from the study. Injury data were obtained from the Medical Health System Management Analysis and Reporting Tool (M2), a centralized data repository that captures data input into the Military Health System’s electronic medical records. A published MSKI taxonomy used in US military MSKI research was used to identify International Classification of Diseases (10th Revision, Clinical Modification) codes corresponding to MSKIs (Hando et al., 2023). Subsequent encounters for the same injury were not counted. The trainees were surveilled for the period corresponding to their 8-week training, and those who suffered any MSKI were recorded. For this analysis, the classification matrix was used to further classify participants as sustaining a lower extremity MSKI (yes/no). Only a small sample of individuals had available data, which resulted in 156 participants between October 2017 and April 2020. Because very few females were enrolled in Special Warfare training at the time, there were no females included in our sample.

### Movement assessment

Within 3 days before the start of the course, kinematic screenings were administered. Eight synced Blackfly/FLIR GigE cameras (50 frames per second) were positioned circumferentially above the participant in a rectangular room measuring 6 m × 6 m and 3 m in height with green screen flooring. Each of the 156 trainees performed the following kinematic movements directed by certified athletic trainers: 1) squat, 2) countermovement jump, (3–4) single-leg squat (each leg), (5–6) lunge (each leg), (7–8) side-lunge (each leg), (9–10) single-leg jump (each leg). Each movement was processed using a validated markerless biomechanics system (SwRI ENABLE™ v.1.0) (SWRI, 2023). We measured six degrees of freedom (DOFs) for the pelvis (3 translation, three rotational), three DOFs for the torso (3 rotational), three DOFs for the shoulders (rotational), three DOFs for the hips (3 rotational), three DOFs for the knees (rotational), two DOFs for the ankles (flexion/extensions, inversion/eversion), and one DOF for the elbows (flexion/extension). In total, 33 DOFs were measured for each individual and for each movement.

### Data analysis

The 33-degree-of-freedom full-body kinematic data obtained from ENABLE™ was analyzed in the aggregate for multiple movements and is referred to herein as an individual’s kinematic profile or biomechanical movement pattern. Each joint angle (or joint displacement in the case of the pelvis) vs. time curve was normalized to 100 evenly spaced time points by two different approaches: 1) by time (start to finish of the movement), and 2) by identifying a starting point of the movement and moving forward 3 s in time, which was found to be sufficient to fully capture each movement for each participant. The first normalization technique identifies relative differences in magnitudes at particular parametrically corresponding points of a movement but is insensitive to time, whereas the second normalization technique is sensitive to the time of a movement but can introduce artificial extrapolations at the end of the data trace if a participant finishes the movement much faster than 3 s. Used in tandem, the two approaches may be complementary (Raffalt et al., 2019). The two normalization techniques across 10 movements created 20 individual kinematic traces for each trainee. Therefore, each trainee’s kinematic profile for a particular movement (referred to here as a kinematic trace) contained 3300 points (33 degrees of freedom x 100 points).

**FIGURE 1:**
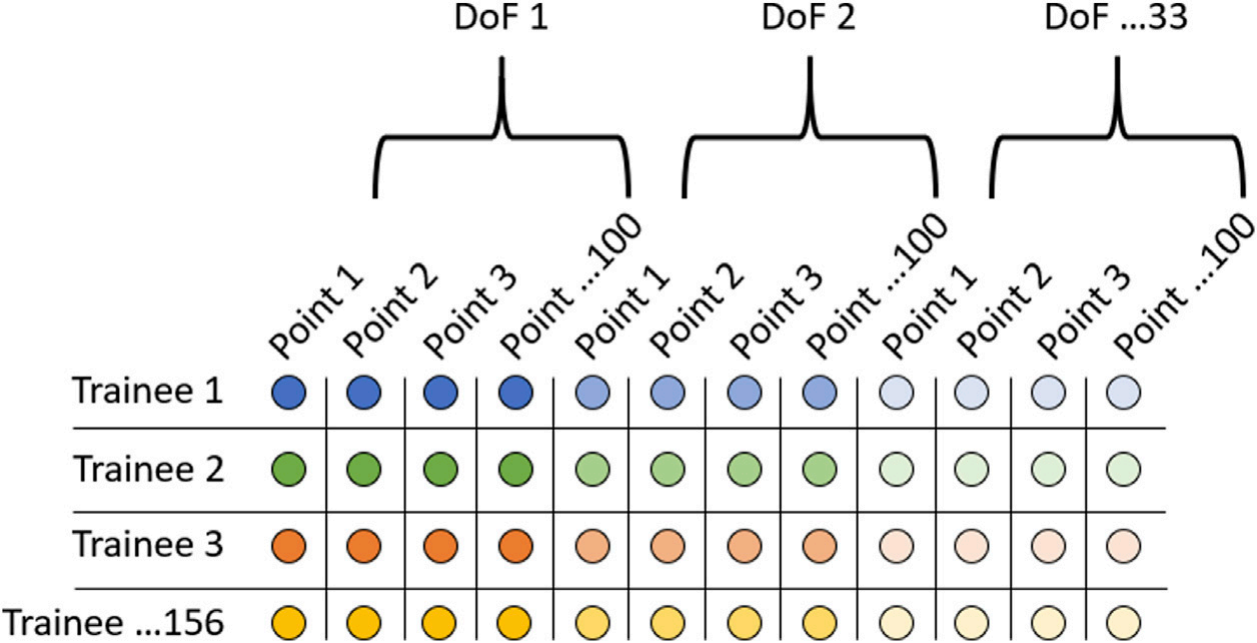
Data organization when performing principal component analysis. Rows represent each of the 156 trainees, and each column represents 1 of 3300 points in the kinematic trace. This was performed for each of the 20 kinematic traces (10 movements x 2 normalizations).

**FIGURE 2:**
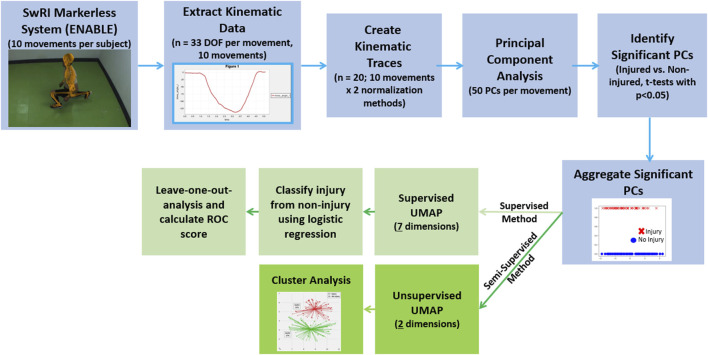
Analysis pipeline for both the unsupervised (dark green) and semi-supervised (light green) approaches. The portions of the workflow outlined in blue are common between both the semi-supervised and supervised analyses.

Principal component analysis was performed separately on each of the 20 kinematic traces across all 156 trainees (Figure 1). For each trace, only the first 50 PCs were kept as anything beyond these explained less than 0.5% of the variance and was deemed too prone to noise error from the markerless motion capture system. Two-tailed t-tests were performed on each of the principal components to test for significant differences between injury and non-injury trainees (*p* < 0.05). The significant PCs from each movement were aggregated and served as input to a semi-supervised and supervised analysis pipeline to test the validity of using full-body kinematic data to assess injury risk (Figure 2).

In the semi-supervised approach (the ‘semi’ is attributed to only using significant PCs), the significant PCs were embedded into 2-dimensional Cartesian space via unsupervised Uniform Manifold and Projection (UMAP) (McInnes et al., 2018). UMAP is a non-linear dimensionality reduction technique and is used in conjunction with the linear PCA to 1) introduce non-linearity to the method, which may detect trends not observable with linear analysis, and 2) further reduce the dimensionality of the PCs, which helps with both supervised learning and visualization (clustering) analysis. The embedded data were then clustered via Gaussian Mixture Models (Reynolds et al., 2009), which is similar to K-Means clustering but does not require that the clusters are circular. The number of clusters was decided via calculating a K-Means silhouette coefficient for 2–10 clusters and selecting the highest score (Shahapure and Nicholas, 2020). The silhouette coefficient is a measure of how close each point in one cluster is to points in the neighboring clusters. A higher score means the clusters are more closely packed, and thus the highest silhouette coefficient calculated denotes the optimal number of clusters to use.

In the supervised approach, supervised UMAP (performed on the significant PCs) was combined with logistic regression (LR) to classify injurious from non-injurious participants. The performance of the model was evaluated via leave-one-out analyses where 155 of the participants were used to train the supervised UMAP embedding and subsequent LR algorithm. The left-out trainee was then passed through the trained UMAP embedding and then classified via the trained LR. The process was repeated for each of the 156 participants. A receiver operating characteristic curve (ROC) was generated and an associated area under the curve (AUC, which is a measure of separability between two classes, or how well the prediction algorithm can distinguish injury from non-injury, in the present study) was calculated. Values above 0.7 are deemed useful for prediction (Mandrekar, 2010). An optimization procedure was performed to identify the best combination of UMAP components, UMAP neighbors, and the LR regularization parameter (C in the sci-kit learn implementation (Pedregosa et al., 2011; Virtanen et al., 2020)). For each participant, prior injury data (any MSKI), which has been shown to be a strong predictor of MSKI during military training (Rhon et al., 2018), was available. As such, the supervised analysis was repeated two more times to 1) only include prior injury data (logistic regression only), and 2) kinematic data + prior injury data. It should be noted that UMAP is a stochastic algorithm. As opposed to fixing the random seed, each analysis was performed 15 times to test if the results were sensitive to random number generation for both the supervised and semi-supervised methods.

## Results

Of the 156 trainees, 48 suffered a lower-body injury. Of these 48 lower body injuries, all were considered overuse injuries that included two stress fractures and two sprains/tears (Table 1).

**TABLE 1:** Injury region and type using Hando et al.’s classification matrix (Hando et al., 2023). 48 total lower extremity injuries occurred in the 156 trainees tracked in this study.

Body region 1 (all categories)		
	Lower Extremity	48
	Spine and Back	8
	Upper Extremity	7
	Head and Neck	3
	Torso	0
	Other	0
Body Region 2 (Top Eight Categories)		
	Knee	19
	Leg Other	15
	Lower Leg	13
	Lumbar Spine	7
	Foot/Toe	6
	Shoulder	6
	Hip	5
	Ankle	3
Injury Types (Top Six Categories)		
	Overuse/Non-Specific	58
	Stress Fracture	2
	Sprain/Joint Damage	1
	Strain/Tear	1

Of the 1000 principal components tested in this study (50 PCs in each of the 20 movements), 53 were found to be significant (Table 1). All movements yielded significantly different PCs. The lunge movements yielded significant PCs that contained the most variance explained, whereas the countermovement jump had the least.

**FIGURE 3:**
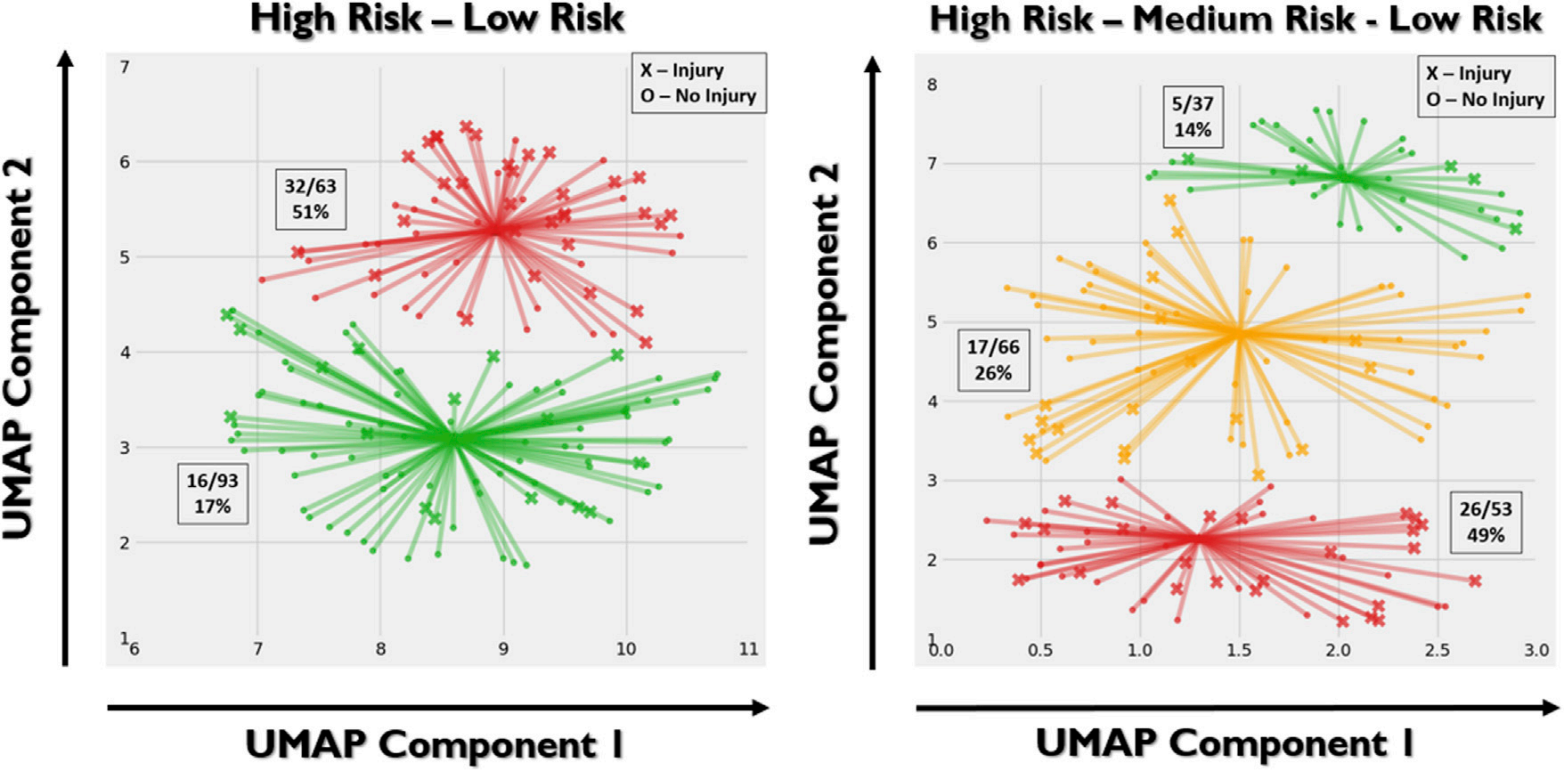
Left: two-group gaussian mixture model clustering. X’s represent injury and O’s represent no injury. Right: three-group clustering. “High-risk” (red), “medium-risk” (yellow), and “low-risk” (green) clusters were defined based on the relative portion of the trainees suffering an MSKI. Based on the stochastic nature of UMAP, each implementation may yield slightly different results even with the same hyperparameters. As such, there is discrepancy between the UMAP embeddings between the two and three group cluster analyses. The *X* and *Y* axes represent the two UMAP variables that the PCA variables were compressed to. The value of each UMAP component is unimportant, whereas the spatial relationship between the points is important.

In the semi-supervised analyses, 2 clusters were selected via the highest silhouette coefficient (0.48). However, *in lieu* of previous work using clustering (Bird et al., 2022), we also implemented 3 clusters (silhouette coefficient = 0.43). In the two-group cluster semi-supervised analysis (Figure 3, left), 51% of the trainees in one of the clusters suffered an MSKI (which we have named the “High Risk” cluster), whereas only 17% of the trainees in the other cluster suffered an MSKI (which we have named the “Low Risk” cluster). The odds ratio was 4.97 (*p* < 0.0001) between these two clusters. In the three-group cluster semi-supervised analysis (Figure 3, right), 49% of the trainees in the “high-risk” cluster suffered an MSKI, 26% of the trainees in the “medium-risk” cluster suffered an MSKI, and only 14% of the trainees in the “low-risk” cluster suffered an MSKI. The repeated analyses had little effect on the clusters.

**FIGURE 4:**
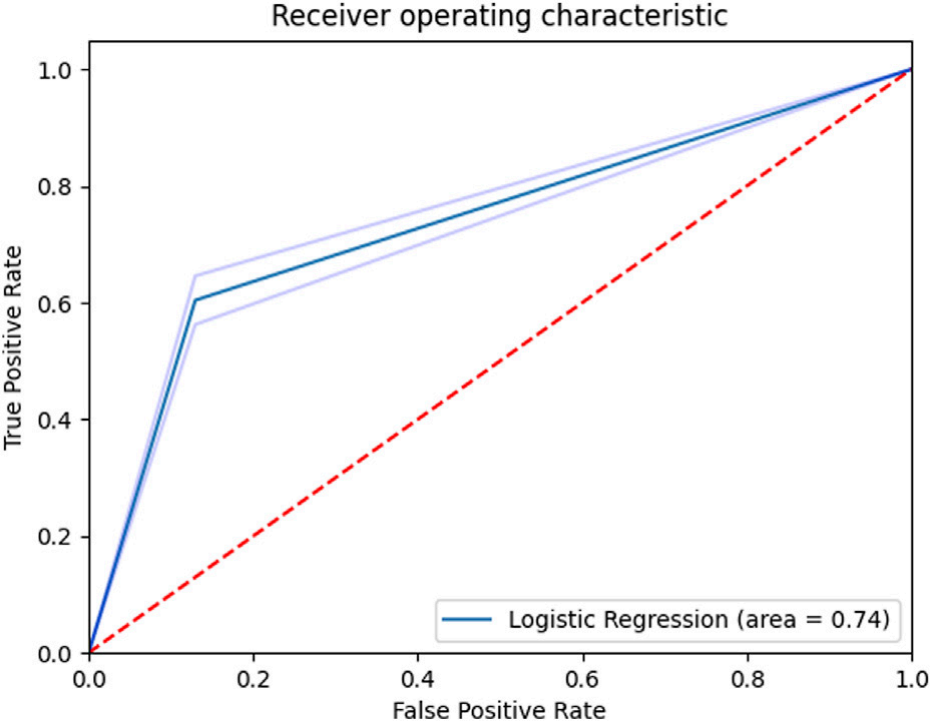
Receiver operating characteristic curve for the supervised classifier (kinematics only). Shaded regions denote ± 1 standard deviation.

**FIGURE 5:**
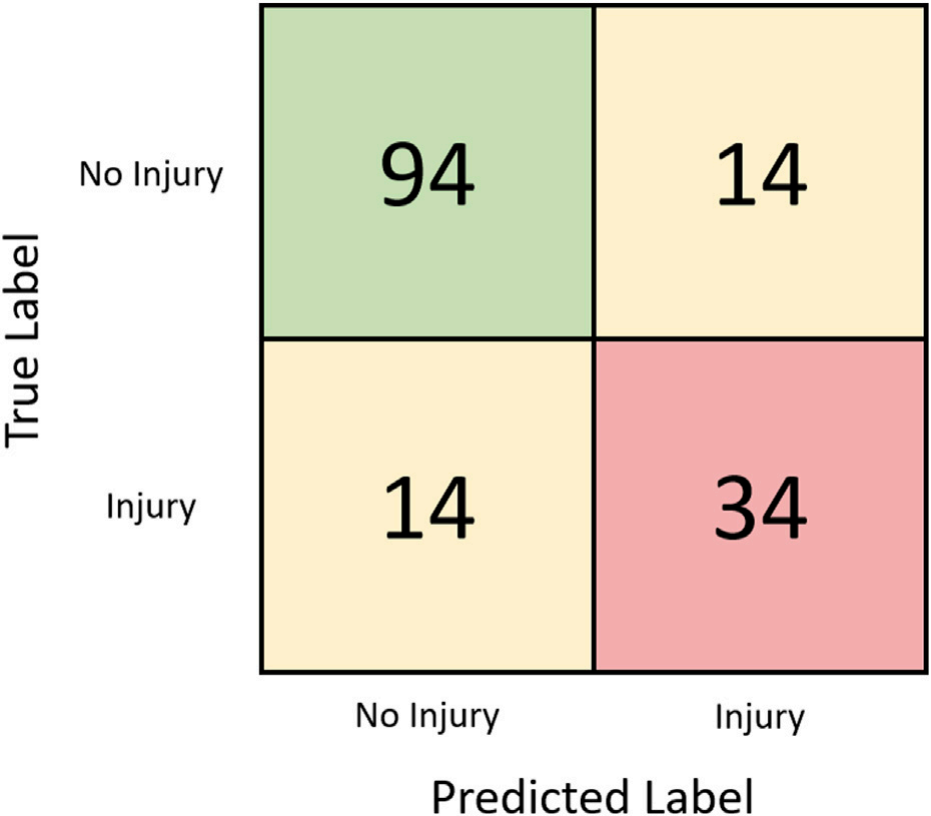
Confusion matrix for the kinematic-only supervised classification task assessed via leave-one-out analysis with the best run (AUC = 0.79).

In the supervised analysis that only included kinematic data, seven UMAP components with the number of neighbors set to 6, and a C value of one was found to be the most optimal combination of hyperparameters in classifying injurious and non-injurious trainees. Using these optimized parameters, an AUC score of 0.74 ± 0.02 (range: 0.70–0.79) was produced (Figure 4 with the best confusion matrix shown in Figure 5). Using the same optimized parameters and the inclusion of prior injury data, an AUC score of 0.75 ± 0.02 (range: 0.71–0.80) was produced. Prior injury data as the sole predictor variable with logistic regression produced an AUC score of 0.62 ± 0.03 (range: 0.57–0.67). The stochastic nature of UMAP had a minor effect on the results (illustrated by the range of AUCs).

## Discussion

The purpose of this study was to investigate whether an individual’s biomechanical movement pattern is indicative of injury risk. The novelty in our study was the approach taken to assess biomechanical movement. Instead of assessing univariate variables at key events (e.g. maximum knee flexion), the entire waveforms for 33 degrees of freedom in the body were analyzed in aggregate using dimensionality reduction techniques. Moreover, several functional movements were analyzed and considered in tandem in the development of both an unsupervised and supervised approach. Considering the time-dependent movement of multiple joints across the body during particular movements, it was found that a semi-supervised analysis naturally clustered individuals based on movement patterns that were indicative of injury-risk. In a supervised approach, we observed that a binary injury classification algorithm may be possible from kinematics alone. An AUC of 0.74 is a promising result and may have implications for identifying MSKI risk in vulnerable populations, such as high-school and collegiate athletes, military Service members, first responders, and others. If the results herein can be repeated and improved upon in a much larger, controlled study, a simple kinematic screening test could be implemented as a means to identify at-risk individuals.

Although other studies that have used biomechanics to assess injury risk primarily focused on univariate measures, they demonstrate a perceived strength in result interpretably. For example, Bird et al. were able to show that a short breaking phase and propulsive phase during the countermovement jump was associated with a lower risk of injury in marine officer candidates (Bird et al., 2022). McHugh et al. report high peak propulsive power in the “above average” group for NCAA athletes (McHugh et al., 2021) during the countermovement jump. In another similar study, Rauch et al. demonstrate that NBA basketball players can be clustered and significantly differentiated based on hip flexion during the downward phase of the countermovement jump (CMJ) (Rauch et al., 2020). Indeed, the CMJ is a popular movement for screening, although we found this movement to be the least indicative of injury risk (lowest variance explained in the significant PCs). These studies, among others, demonstrate the benefits of using distinctly chosen predictor variables because these variables are understandable, and they can be interpreted as to what they might be revealing in military trainees (or athletes). Therefore, a criticism against full-body data analyses that use variable, or dimension, reduction methods such as principal components analysis of raw kinematic data could be that the results are difficult to interpret as each PC contains movement information across multiple degrees of freedom (in this study, 33). However, this approach coupled with rigid body dynamics and visualization software makes interpretability possible. To this end, we provide the following explanation on how this can be accomplished.

**FIGURE 6:**
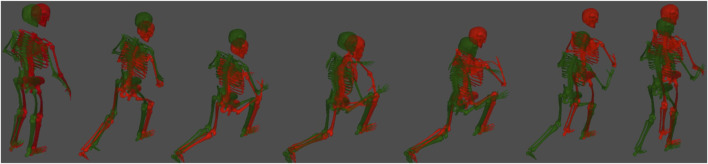
Time series of a side lunge (right leg) for both the plus and minus 1 standard deviation of a randomly selected principal component. These models are constructed in OpenSim (native output of ENABLE™) by taking the average kinematics across all participants and perturbing the average motion by a weight factor multiplied by a principal component. The weight factors shown here are plus 1 (green) standard deviation (in this particular PC, plus one was associated with non-injury) and a minus 1 (red) standard deviation, which was associated with injury.

Using OpenSim (which is the native output of ENABLE™), the principal components that are significant between injury and non-injury participants can be isolated and visualized by perturbing an “average” model with the plus and minus 1 standard deviation of that particular principal component. This type of analysis has substantial implications for training and intervention strategies, as it allows significantly different movement patterns between groups to be visually scrutinized. As a demonstration of this capability and motivation for future work, we visually compared the plus and minus 1 standard deviation of a randomly selected principal component that was significantly different between groups (right side lunge PC#7 that explained 5% of the variance). We found that on average, those that went on to suffer a lower-extremity MSKI performed the side lunge faster than their non-injured counterparts (Figure 6). Moreover, the injured group tended to take a smaller backward step during the initial eccentric phase of the movement. While these observations are not predictive of injury risk in isolation, they provide additional information to researchers and trainers that may help guide actionable interventions. Considering all of the significant PCs, a trainer/practitioner may find insightful differences between each group, such as balance or strength issues and use this information to design intervention strategies aimed at risk mitigation.

The machine learning algorithms implemented in this study were PCA, UMAP, and logistic regression. While the results are positive, we do not believe the results are specific to the choice of algorithms. PCA is a statistical, easy-to-implement algorithm that reduces the dimensionality of the data. However, it is not the only choice. KPCA, autoencoders, diffusion maps, and other techniques can reduce the burden of high-dimensional data. The benefit of using PCA is that the 1-to-1 linear inverse mapping presents an opportunity to readily visualize the movements associated with each PC, as discussed above. UMAP was selected to further reduce the dimensionality of the data and provide non-linearity that PCA and logistic regression do not provide. Again, other algorithms are available, but we have found that UMAP is a robust algorithm for dimensionality reduction and is advantageous in that both supervised and unsupervised embeddings are allowable. Moreover, UMAP preserves global data structure such that the relative location of a particular point (and cluster) is meaningful in relation to others. This is evident in the two-cluster analysis in Figure 3. Individuals in the high-risk cluster that were spatially closer to the low-risk cluster tended to be non-injurious, which suggests a “medium-risk”. Unsurprisingly then, the semi-supervised three-cluster analysis naturally yielded an intermediate “medium-risk” zone. It should be noted that we observed a minor effect on the random number generation within UMAP, which yielded AUC values between 0.70–0.80. This is likely due to the small sample size as a single change (1 out of 156) in the predicted class changed the AUC by 0.01. Logistic regression is a common binary classifier, but many other options exist to perform binary classification. We find it encouraging that our first choice of algorithms performed well, and perhaps improvement can be made with a more thorough investigation of alternative algorithms.

There were several limitations of this study. The sample size of 156 was low, and it is unknown if the same predictive power would be obtained from a larger military population. Moreover, due to the small sample size, the ability to predict specific lower extremity injuries (Table 2, Body Region 2) was not investigated. The participants were spread over several cohorts, and therefore not every participant performed the exact same training regimen (although, this may be considered a strength, since this could indicate algorithm generalizability). Age and BMI, and fitness measures were not available for this analysis, but they’ve also been shown to be predictive of injury risk (Rappole et al., 2017; Rhon et al., 2018; Hollander et al., 2020; Butler et al., 2022; Hando et al., 2022). Also, while not necessarily a limitation of this study, force plate data were not available, and the inclusion of kinetic data would provide a more complete biomechanical assessment of an individual and may improve the predictive power of the algorithms used in this study (Guess et al., 2020; Thomas et al., 2022).

**TABLE 2:** The number of significant principal components identified for each movement and their respective cumulative variance explained.

	Number of significant PCs (*p* < 0.05)	Total variance (%) explained
Left Lunge	4	17
Right Lunge	3	14
Squat	8	13
Right Side Lunge	9	10
Left Single Leg Squat	7	10
Left Single Leg Jump	6	10
Right Single Leg Squat	5	10
Right Single Leg Squat	5	7
Left Side Lunge	4	5
Countermovement Jump	2	2

In conclusion, we found that full-body kinematics may be predictive of lower extremity MSKI in male trainees that undergo an 8-week Air Force Special Warfare preparatory course. The analysis was performed with data collected from markerless motion capture (ENABLE™). The findings from this preliminary study warrant further investigation and may lead to advanced methods to identify individuals at-risk for injury in military, sport, and healthcare. If at-risk individuals can be readily identified with quick, non-invasive functional movement screens, then tailored interventions, such as specific exercise programs, may be investigated in a controlled study to test its efficacy in reducing the incidence of injury.

## Data Availability

The datasets presented in this article are not readily available because the data comes from Air Force trainees and we have been given special permission to use it. Requests to access the datasets should be directed to cody.butler.3@us.af.mil.
